# Rheumatic Diseases and the Heart

**Published:** 1989-05

**Authors:** Paul Dieppe


### REFERENCE

BURCHARTH, F., BALLAN, A., KYLBERG, F. *et al* (1986) The colostomy plug: a new disposable device for a continent colostomy. *Lancet* **i**, **ii**, 1062–1063.

### Book Review

**Rheumatic Diseases and the Heart,** by John A Cosh and John V. Lever Springer Verlag 250pp 1988

This book is a tribute to the excellent work of its senior author John Cosh, who combined the skills of physician, cardiologist and rheumatologist so effectively for so many years. His co-author is a pathologist, and the marriage is successful. One of the main themes of the book is the common pathogenesis of cardiac and joint lesions in the rheumatic diseases.

The diseases covered include rheumatic fever, infective endocarditis, rheumatoid arthritis, SLE, systemic sclerosis, the sero-negative spondarthritides and a variety of miscellaneous conditions. In each chapter a general introduction is followed by a more detailed description of the pathology and clinical features as they affect the heart. The text is liberally spiced with pictures, tables and case illustrations. Its basis in long experience, careful observation and the skills of a superb clinican and pathologist are apparent on every page.

The cardiac manifestations of rheumatic disease illustrate the systemic nature of many 'joint' diseases. John Cosh has contributed extensively to the revolution in rheumatology, which has elevated it from a minor specialty related to orthopaedics and physical medicine to a major branch of general medicine. This book helps explain why all physicians should know some rheumatology, and why all rheumatologists must be good general physicians. It also illustrates one of the main fascinations of the rheumatic diseases: i.e. the insight they provide to the understanding of inflammatory disorders and organ pathology in general. As this monograph explains, rheumatoid arthritis is a fascinating 'model' of multisystem pathophysiology. It is also a major cause of pain, physical disability and emotional problems. The total management of the rheumatoid patient calls on all the expertise of a physician and carer.

The authors indicate that their book assumes no special knowledge of either rheumatology or cardiology. They hope it will be useful to specialists in these and other branches of medicine. I am sure they are right on both points, and a nicely produced book of this sort, spanning different disciplines, is a pleasure to read. It also provides extensive, up to date references (including one 1989 reference in a 1988 book!), which will make it a very useful reference book. The only reservation relates to its price and its market. I wonder who will want to spend £50 on a specialist monograph of this sort, which has limited value, even for a cardiologist or rheumatologist.

I hope many people do opt to buy this book. John Cosh, who served the South West so well, and John Lever deserve it.

Paul Dieppe

